# Cybersecurity in Healthcare: New Threat to Patient Safety

**DOI:** 10.7759/cureus.83614

**Published:** 2025-05-06

**Authors:** Bakheet Aldosari

**Affiliations:** 1 Health Informatics, King Saud Bin Abdulaziz University for Health Sciences, Riyadh, SAU

**Keywords:** cybersecurity, digitalization, healthcare system, healthcare technology, patient care

## Abstract

The rapid integration of technology into healthcare systems has brought significant improvements in patient care and operational efficiency, but it has also introduced new cybersecurity challenges. This manuscript explores the evolving landscape of cybersecurity risks in healthcare, with a focus on their potential impact on patient safety and the strategies to mitigate these threats. The rise of interconnected systems, electronic health records (EHRs), and Internet of things (IoT) devices has made safeguarding patient data and healthcare processes increasingly complex. Notable cyber incidents, such as the Anthem Blue Cross breach and the WannaCry ransomware attack, highlight the real-world consequences of these vulnerabilities. The review also examines emerging technologies like AI, cloud computing, telehealth, and wearables, considering their potential benefits and security risks. Best practices for improving healthcare cybersecurity are discussed, including regulatory compliance, risk assessment, data encryption, employee training, and incident response planning. Ultimately, the manuscript emphasizes the ethical responsibility of healthcare organizations to prioritize cybersecurity, ensuring a balance between innovation and security to protect patient data, uphold regulatory standards, and maintain the integrity of healthcare services.

## Introduction and background

Over the past decade, healthcare technology adoption has increased exponentially, creating numerous benefits and presenting unique challenges, particularly regarding cybersecurity [1]. Given how heavily digital systems rely on each other and play an essential role in protecting patient safety and safeguarding sensitive health data [2]. With increased reliance on these practices and an ever-increasing reliance on them being implemented across organizations, cybersecurity practices play a crucial role in maintaining patient safety while safeguarding sensitive health records from being breached or leaked out in any form [3-4]. Cyberattacks against healthcare institutions are an alarming trend that must be dealt with immediately [5]. Such cyberattacks compromise system and data security and can pose immediate threats to human life and well-being [6]. 

The healthcare sector faces challenges that require a comprehensive yet tailored cybersecurity solution [7]. One major challenge facing the healthcare industry is cyberattacks of various sorts. Malware may endanger patient privacy, while distributed denial of service (DDoS) attacks disrupt patient care - the risks are manifold [8]. Ransomware poses great threats, as losing medical data could compromise lives directly [9]. Each healthcare organization, whether a hospital, clinic, or private practice, is directly responsible for protecting its systems and patient data, following government standards, and needs more resources for investing in comprehensive cybersecurity measures. This vulnerability extends to outdated technologies, inadequate cyber risk training for staff, and the security vulnerabilities of medical devices connected to the Internet of Things (IoT) [9]. As cyberattacks continue to escalate, the demand for cybersecurity professionals is on the rise, but there needs to be more skilled experts to tackle these threats effectively [10]. Looking ahead, the healthcare industry must strive to enhance its cybersecurity posture. This requires a proactive approach that prioritizes up-to-date technologies, rigorous training for healthcare staff, and robust safeguards for medical devices. Collaboration between healthcare, technology, and cybersecurity experts will be essential in devising innovative solutions to the evolving challenges posed by cyberattacks [9,11,12].

## Review

Rationale

In an age of rapid technological advancement, the healthcare sector stands at the intersection of innovation and vulnerability. The adoption of digital technologies has revolutionized patient care but has also exposed critical weaknesses in cybersecurity [13]. Amidst this dynamic landscape, cyberattacks' frequency, sophistication, and impact on healthcare institutions have surged. Interconnected devices and the rapid proliferation of IoT technologies have expanded the attack surface, with anesthesia and intensive care units (ICUs) serving as particularly vulnerable arenas. The potential for malevolent actors to manipulate interconnected medical devices and exploit network access points presents an alarming threat, necessitating a thorough investigation [14,15]. The consequences of successful cyberattacks extend far beyond compromised data. Patient safety hangs in the balance, with breaches leading to altered treatment plans, erroneous monitoring, and disruptions to critical care devices. The dire implications of interconnected anesthesia and ICU devices underscore the immediate need for fortified cybersecurity measures [16]. The COVID-19 pandemic, while accelerating telemedicine and remote work, has also exposed vulnerabilities in cybersecurity practices. This unprecedented shift in healthcare delivery has introduced new entry points for cyberattacks, magnifying the risk to patient well-being and the integrity of healthcare operations [9,10]. Compounding these challenges is the chronic underfunding of cybersecurity in healthcare, leaving the sector ill-equipped to counter evolving threats. The monetization of stolen healthcare data and the influx of technically insecure IoT devices have amplified the urgency for investment in cybersecurity infrastructure [2]. In this complex landscape, a multidisciplinary approach becomes imperative. Collaboration between healthcare, technology, and cybersecurity domains is essential to devise innovative solutions and policy changes that safeguard patient safety and data integrity. This comprehensive review thoroughly investigates, connecting the dots between the evolving landscape of cyber threats, patient safety imperatives, technological progress, financial shortcomings, and interdisciplinary teamwork. Its primary objective is to highlight the necessity for robust cybersecurity protocols aligning with digital healthcare's transformative impact. By meticulously examining these intricate interplays, the review emphasizes the urgency of proactive measures to safeguard sensitive healthcare systems [9,16,17].

Overview of cybersecurity in healthcare

The healthcare landscape has become increasingly vulnerable to cyber threats in an era of digitized medical records, interconnected devices, and telehealth platforms. Technology integration has facilitated efficient healthcare delivery, but it has paved the way for nefarious cyber activities that jeopardize patient safety and confidentiality. This article delves into the multifaceted realm of cybersecurity within the healthcare industry, exploring its definition, historical evolution, current challenges, and profound implications for patient welfare [9,17]. Cybersecurity in healthcare settings encompasses the set of practices and measures to safeguard digital systems, networks, and data from unauthorized access, disruption, or manipulation. In the healthcare domain, cybersecurity extends beyond traditional IT concerns; it encompasses the protection of medical devices, electronic health records (EHRs), telehealth platforms, and the intricate web of interconnected systems that form the backbone of modern healthcare delivery [2,16]. Unlike other sectors, healthcare cybersecurity carries distinctive implications due to the sensitivity of the data at stake. Patient health records, treatment plans, and medical histories constitute a treasure trove for hackers seeking to exploit vulnerabilities for financial gain, identity theft, or even the manipulation of treatment protocols. Thus, cybersecurity in healthcare involves not only shielding digital infrastructure but also preserving the integrity of patient care processes [9,18].

A staggering increase in the frequency and complexity of cyberattacks has marked the evolution of cybersecurity challenges in healthcare. While the healthcare sector previously relied on isolated systems and paper records, the transition to electronic records and interconnected devices has opened new avenues for malicious actors to exploit [19]. Initially, cybersecurity threats were viewed primarily through the lens of data breaches, where patient records were compromised for financial gain. However, the landscape has since expanded to encompass more insidious threats, such as ransomware attacks, where hackers seize control of critical systems and demand hefty ransoms for their release [20]. What sets healthcare cybersecurity apart is its intersection with patient safety. Malicious interference with medical devices, such as pacemakers and insulin pumps, can have life-threatening consequences [21]. Furthermore, the COVID-19 pandemic unveiled the vulnerabilities of hastily implemented telehealth platforms, leading to attacks targeting remote patient care systems. This evolving threat landscape requires a comprehensive and adaptive cybersecurity framework that safeguards data and lives [10,22]. As the healthcare sector grapples with the ever-expanding cyber threat landscape, cybersecurity reveals a mixed tapestry of progress and vulnerabilities. On the one hand, healthcare organizations have recognized the significance of cybersecurity and invested in technical measures such as encryption, multi-factor authentication, and intrusion detection systems. These measures fortify the digital perimeter, deterring many potential attacks [23]. However, the reality remains that healthcare cybersecurity efforts are often misdirected. While technological solutions are crucial, the human element must be considered. Phishing attacks, social engineering, and human error are potent vectors for cyber incidents. Moreover, the proliferation of IoT medical devices with inadequate security measures has introduced a host of entry points for cybercriminals [24]. The impact of inadequate cybersecurity on patient safety is profound. Beyond data breaches and financial implications, patient lives are at stake when hackers exploit vulnerabilities in medical devices or disrupt clinical workflows. Vulnerabilities in telehealth platforms undermine the essence of remote healthcare delivery, eroding patient trust and impeding access to care [25]. The intricate interplay between technology, data, and patient safety underscores the imperative of robust cybersecurity in the healthcare sector. The evolution of cyber threats demands a holistic approach that integrates technical solutions with comprehensive training, risk management, and collaboration across healthcare stakeholders. As healthcare increasingly relies on digital tools, addressing cybersecurity challenges is not merely a matter of protection but a vital component of ethical and responsible patient care [26].

Types of cybersecurity threats in healthcare

In the intricate healthcare cybersecurity landscape, myriad threats loom, each posing unique challenges to patient safety and data integrity [27]. This section delves into the five prominent categories of cybersecurity threats within the healthcare industry, shedding light on the nuances, potential consequences, and strategies for mitigation. The first is malware, a malicious software designed to infiltrate and disrupt computer systems, which has become a formidable adversary in healthcare cybersecurity [28]. The subcategory of ransomware attacks has garnered particular notoriety, involving the encryption of critical data and a demand for ransom in exchange for its release. These attacks paralyze healthcare institutions, jeopardizing patient care, delaying treatments, and potentially leading to life-threatening situations [29]. The aftermath of a successful ransomware attack extends beyond financial loss; patient records and treatment plans can be held hostage, impacting clinical decision-making and patient trust. Mitigation strategies involve regular system patching, up-to-date antivirus software, and robust backup protocols to minimize the impact of potential attacks [30]. Data breaches remain a pervasive concern in healthcare cybersecurity, often stemming from unauthorized access to patient records [31]. The theft of personal health information exposes patients to identity theft, fraud, and compromised medical history confidentiality. These breaches erode patient trust and carry legal and financial repercussions for healthcare organizations [32]. Prevention measures entail stringent access controls, robust authentication mechanisms, and regular security audits to identify and rectify vulnerabilities. Moreover, encryption and tokenization techniques can render stolen data unusable, providing an added layer of protection [33]. Exploiting the human element, social engineering and phishing attacks target employees through deceptive emails, messages, or phone calls, tricking them into revealing sensitive information or inadvertently granting unauthorized access [34]. These attacks thrive on manipulating human psychology and capitalizing on the chaotic healthcare environment. To counteract these threats, healthcare organizations must prioritize cybersecurity education and training [35]. Employees must be equipped to identify phishing attempts, validate requests for information, and exercise caution when engaging in unsolicited communication.

The increasing integration of medical devices into healthcare networks introduces a novel avenue for cyber threats [36]. Medical device vulnerabilities encompass the potential for hackers to gain control of devices such as pacemakers, infusion pumps, and imaging systems. These vulnerabilities can have dire consequences, including altered treatment delivery or life-threatening device manipulation [37]. To mitigate these risks, healthcare institutions must collaborate with device manufacturers to ensure robust cybersecurity measures are embedded in the design and operation of medical devices. Continuous monitoring, timely patching, and strict segmentation of device networks from critical systems are essential safeguards [2]. While external threats draw significant attention, insider threats and employee negligence are equally significant [38]. Inadvertent actions by employees, such as downloading malicious software or mishandling sensitive data, can inadvertently open pathways for cyberattacks. Intentional actions by disgruntled employees can also result in data breaches or system disruptions.

Healthcare organizations must foster a culture of cybersecurity awareness, encouraging employees to prioritize patient safety through vigilant practices [39]. Implementation of role-based access controls, periodic cybersecurity training, and robust monitoring of employee activities can mitigate the risks posed by insider threats. The spectrum of cybersecurity threats in healthcare is multifaceted, encompassing a wide array of vectors that demand diverse and adaptive mitigation strategies. The intertwined nature of technology, data, and patient welfare necessitates a comprehensive approach that aligns technical measures with human awareness and collaborative efforts [40]. As the healthcare industry continues to innovate and evolve, a steadfast commitment to cybersecurity remains pivotal to upholding patient safety and data integrity.

Consequences of cybersecurity threats on patient safety

The intricate intersection of healthcare and technology has birthed a new era of patient care, but with it comes a range of unprecedented cybersecurity threats [41]. This section delves into the cascading consequences of cyber threats on patient safety, underscoring the far-reaching implications these breaches and attacks have on the individuals seeking care and the healthcare organizations responsible for their well-being. Cybersecurity threats pose two immediate and alarming patient outcomes: compromised data and privacy violations [42]. Once considered private within healthcare institutions, personal health information now risks unauthorized access and possible exposure; this compromise damages trust between patients and healthcare providers and exposes patients to identity theft, financial fraud, and psychological trauma [43].

Consequences extend beyond individuals to healthcare organizations, which could face regulatory fines or lawsuits for failing to protect patient data [44]. To defend themselves from this threat, healthcare institutions must prioritize robust encryption, access controls, and data monitoring protocols to guarantee patient privacy. Cyberattacks frequently disrupt healthcare operations and services, with cybercriminals rendering critical systems, medical devices, and communication networks inoperable [45]. Such disruptions can paralyze hospital workflows, delay patient care delivery, compromise emergency services provisioning capabilities, and undermine clinical decision-making, putting patient safety at risk. Healthcare organizations must implement preventive measures, including redundancy, network segmentation, and disaster recovery plans, to safeguard essential services and patient care against cyber incidents [46].

Because healthcare technologies are intertwined, any breach can have far-reaching repercussions for patient care and treatment. Cyberattacks may alter treatment plans, modify electronic prescriptions, or manipulate diagnostic data, with potentially life-threatening results for patient safety due to cyber intrusions [47]. Healthcare institutions must implement stringent access controls and audit trails and invest in secure communication platforms that protect medical information and treatment processes to provide safe patient care [48]. Healthcare organizations suffering cyberattacks face considerable financial implications following cybersecurity incidents, with remediation efforts, data recovery costs, legal fees, and potential regulatory fines all straining limited resources [49]. Furthermore, affected patients could file lawsuits seeking compensation for compromised data or disruption to services that impact them directly. Healthcare organizations looking to prevent financial losses should invest in cybersecurity measures focusing on prevention, early detection, and rapid response to minimize any resulting cyber incidents [50].

Healthcare cybersecurity regulations are constantly shifting, underscoring healthcare institutions' need for compliance and preparedness [51]. They must comply with regulations like the Health Insurance Portability and Accountability Act (HIPAA) and the European Union Regulation General Data Protection Regulation (GDPR) to safeguard patient data and privacy; failure to do so could incur significant fines and legal liabilities for noncompliance. Legal cases from affected patients seeking damages due to cyber incidents further demonstrate the legal quagmire healthcare organizations may face [52]. Cybersecurity threats to patient safety have wide-reaching impacts affecting all healthcare delivery areas, from compromised data and disrupted operations to patient care disruption and financial implications [53]. Given our current technologically driven healthcare landscape, cybersecurity should be seen as a necessary technical task and an ethical imperative to safeguard patients seeking treatment [54].

Emerging trends and challenges in healthcare cybersecurity

As healthcare continues its relentless integration with technology, emerging trends bring opportunities and challenges in equal measure [55]. This section illuminates the evolving landscape of healthcare cybersecurity, highlighting the transformative potential of new technologies and the complex security considerations accompanying them. The IoT has introduced a paradigm shift in healthcare by facilitating the connection of medical devices and equipment to the Internet, enabling real-time monitoring and data sharing [56]. However, the proliferation of connected devices has opened up many security holes. From pacemakers and infusion pumps to MRI machines, connected devices pose security threats by being vulnerable to hacking, manipulation, and unauthorized access [57]. Security for IoT devices must take an integrated approach that includes robust authentication mechanisms, encryption, continuous monitoring, and frequent updates [58]. Manufacturers and healthcare organizations should work collaboratively to embed security into the design and lifecycle of these devices.

Cloud computing has revolutionized healthcare data storage and accessibility, offering scalability and flexibility [59]. However, trusting patient information with third-party cloud providers presents new risks, such as data breaches, unauthorized access, or losing control over sensitive information. Healthcare organizations must understand how to navigate the intricacies of cloud security, emphasizing access controls, encryption, and binding contractual agreements with cloud providers [60]. Achieving an equilibrium between convenience and security is of utmost importance in order to keep patient data safe. Cloud computing has revolutionized data storage and accessibility in healthcare, offering greater scalability and flexibility than its counterparts [61]. However, trusting patient data with third-party cloud providers introduces additional risks, including data breaches, unapproved access, and loss of control over sensitive information. Healthcare organizations must navigate the intricacies of cloud security with care, prioritizing strict access controls, encryption, and contractual agreements with cloud providers to protect patient data while offering convenience for staff members [62]. Striking an optimal balance between convenience and security must always remain a top priority to safeguard patient records from compromise [63].

Artificial intelligence and machine learning (ML) have begun transforming diagnostics, treatment planning, and drug discovery [64]. However, AI-driven technologies demand vast datasets, raising concerns about patient privacy and data security. Moreover, AI systems can be manipulated, leading to erroneous diagnoses or biased treatment recommendations [65]. Mitigating these challenges involves anonymizing patient data, adopting ethical AI practices, and rigorously testing AI systems for vulnerabilities [66]. The transparency of AI algorithms and the ethical handling of patient data are pivotal to fostering trust in AI-powered healthcare solutions. Mobile health applications and wearable devices empower individuals to monitor their health and engage in proactive self-care [67]. However, these devices often collect sensitive health data, leaving users vulnerable to data breaches and identity theft. The blurring lines between personal and medical data intensify the need for robust cybersecurity [68].

Developers of mobile health apps and wearables must prioritize user privacy, implement strong encryption, and ensure secure data transmission. Healthcare organizations should educate patients on the importance of using reputable apps and devices and exercising caution with their health-related data [69]. In conclusion, the evolving landscape of healthcare is marked by promising technological advancements that hold the potential to reshape patient care [70]. However, these innovations come hand-in-hand with intricate cybersecurity challenges. The interconnectedness of devices, the cloud, telehealth, AI, and wearables requires healthcare institutions to prioritize cybersecurity at every level. Striking a delicate balance between embracing technological progress and safeguarding patient safety is the defining task of healthcare cybersecurity in the face of these emerging trends [71].

Current approaches and strategies for healthcare cybersecurity

In the ongoing battle against evolving cyber threats, healthcare organizations adopt multifaceted strategies to fortify their defenses. This section delves into the key approaches employed to safeguard patient data and preserve the integrity of healthcare operations. Regulatory bodies such as the HIPAA and GDPR mandate security standards for protecting patient information [72]. Healthcare organizations must adhere to these frameworks, outlining protocols for data security, breach notification, and privacy safeguards. Compliance ensures legal adherence and cultivates a culture of cybersecurity awareness [73]. Thorough risk assessment forms the cornerstone of effective cybersecurity. Healthcare institutions must systematically identify vulnerabilities and potential threats across their technological landscape [74]. With this insight, they can prioritize resources, implement targeted security measures, and create robust risk management strategies. Regular evaluations ensure ongoing adaptability to emerging threats [75]. Encryption is a formidable defense mechanism against unauthorized access to patient data [76]. Adopting end-to-end encryption ensures that data remains indecipherable to malicious actors even during a breach. Techniques like tokenization replace sensitive data with surrogate values, rendering it useless to cybercriminals. By layering encryption protocols, healthcare organizations create formidable barriers to data compromise [77]. Human error remains a significant vulnerability in cybersecurity. Healthcare organizations recognize the importance of educating their personnel about cybersecurity best practices [78]. Comprehensive training and awareness programs empower employees to identify phishing attempts, avoid social engineering tactics, and navigate the digital landscape responsibly. Cultivating a cybersecurity-conscious workforce fortifies the last defense against breaches [79].

Recognizing the inevitability of cyber incidents, healthcare institutions are bolstering their incident response and recovery strategies [80]. Rapid detection, containment, and mitigation are pivotal to minimizing the impact of breaches. Organizations outline premeditated response plans, detailing roles, communication channels, and steps to remediate breaches and restore operations. Post-incident analysis informs future improvements and fine-tunes response protocols [81]. Healthcare organizations are realizing the value of collective defense against cyber threats [82]. Collaborative platforms facilitate the exchange of threat intelligence, enabling the early identification of emerging risks. Sharing insights on attack vectors, tactics, and vulnerabilities empowers the industry to anticipate threats and devise proactive countermeasures [83]. In the dynamic realm of cybersecurity, a holistic approach is indispensable. Healthcare organizations must seamlessly integrate regulatory compliance, risk management, encryption, employee education, and robust incident response to foster a comprehensive defense posture [84]. As the threat landscape evolves, these strategies form an interconnected web of protection, guarding patient data and ensuring the continuity of quality healthcare services [85].

Notable cybersecurity incidents in the healthcare sector

In one of the largest healthcare data breaches, Anthem Blue Cross suffered a cyberattack that exposed the data of 78.8 million individuals. The breach was caused by a phishing attack, allowing hackers to access sensitive personal and medical information [86]. Another cybersecurity incident was the global WannaCry ransomware attack that caused significant disruption across many sectors, including healthcare [87]. In particular, the UK's National Health Service was severely impacted, disrupting operations and jeopardizing patient care, underscoring healthcare systems' susceptibility to ransomware attacks [88]. The University of California, San Francisco (UCSF) was also targeted with a ransomware attack that targeted their School of Medicine servers, disrupting research operations and patient care systems [89]. Although the UCSF refused to pay the ransomware demand, this incident highlighted the potential risks of compromised medical research and healthcare services [90].

Impact on patient safety and healthcare organizations

The Anthem breach exposed millions of individuals' personal and medical data, leaving them at risk of identity theft and fraud [91]. Compromised records included social security numbers, medical IDs, addresses, and employment details, significantly undermining trust in Anthem's security while damaging its reputation [92]. The WannaCry attack on the NHS resulted in canceled appointments, delayed treatments, and diverting emergency patients to other hospitals, placing patient safety in danger due to compromised systems and clarifying how cyberattacks on healthcare facilities could have life-threatening repercussions [93]. The UCSF ransomware attack was devastating, disrupting crucial medical research and patient care systems and impacting ongoing research projects and medical services [94]. Restoring operations became difficult while impacting ongoing projects or delaying services, showing the broad implications cyberattacks can have on healthcare organizations' ability to deliver quality healthcare services [17].

Lessons learned and best practices

Healthcare organizations must allocate sufficient funds and implement robust cybersecurity measures, including regular security audits, software updates, and employee training, to minimize vulnerabilities and avoid breaches [95]. Encrypting patient data provides another layer of protection in the case of a breach, making it less useful to attackers who do not possess decryption keys [96]. Healthcare organizations should develop and regularly update incident response plans to minimize the effects of cyberattacks, with strategies in place for isolating affected systems, notifying patients of affected attacks, collaborating with law enforcement officials, and cooperating on investigations [97]. Informing healthcare staff of cybersecurity risks and best practices is of utmost importance, while training on phishing awareness can protect employees against falling prey to email-based attacks [19]. Network segmentation can limit lateral movement within an organization's systems and ensure that only authorized personnel can access sensitive patient data [98]. Regularly backing up critical systems and data can aid in recovery after a cyberattack. Organizations should ensure that backups are secure and accessible during emergencies [99]. Healthcare organizations should collaborate with cybersecurity experts and share information about emerging threats and vulnerabilities within the sector [100]. Adhering to healthcare data protection regulations, such as the HIPAA, is crucial. Compliance helps ensure patient data is handled securely, and organizations are prepared for potential audits [101].

Emerging insights

This narrative review offers several noteworthy insights that arise about cybersecurity's effect on patient safety. This review emphasizes the intricate interdependencies between healthcare systems, noting how cyber threats do not just compromise data but can disrupt patient care processes and even lead to potentially life-threatening situations. Furthermore, an ever-evolving threat landscape highlights how vulnerabilities in one area, such as medical devices, can cascade across operations in healthcare and ultimately have adverse impacts on patient well-being. Healthcare cybersecurity has unique implications due to the sensitivity of patient data. The manuscript illustrates this fact by noting how compromised health records not only increase identity theft and fraud risk but can have direct negative implications on care provision; compromised medical devices or altered treatment plans could have serious and immediate repercussions for patients, underscoring how tightly connected cybersecurity and patient safety are. The review takes an emphatic ethical stance toward cybersecurity, stressing its significance as both an ethical imperative and a technical concern. Cyberattacks represent potential harm that must be mitigated to ensure patient safety, data integrity, and continuity of care - this requires healthcare organizations to prioritize cybersecurity measures to protect patient wellbeing, data integrity, and continuity of care. This review discusses emerging trends like IoT, cloud computing, telehealth, AI, and wearables that promise transformative potential in healthcare but introduce new vulnerabilities requiring creative approaches to security. Healthcare continues to advance, necessitating proactive cybersecurity measures that keep up with these innovations and can mitigate emerging risks as healthcare innovates. The narrative review underscores the necessity of collaboration across healthcare, technology, and cybersecurity. Interdisciplinary teamwork is imperative to effectively respond to ever-evolving threats while offering innovative solutions that ensure patient safety. This review found there must be an essential balance between technological progress and patient safety, emphasizing how healthcare technology offers great potential but must be utilized responsibly through robust cybersecurity measures that protect patient data and care processes.

## Conclusions

This review emphasizes the urgent need for healthcare institutions to adopt comprehensive cybersecurity strategies as a core ethical responsibility. As digital innovations like AI, IoT, and telehealth enhance care delivery, they also expose systems to significant cyber threats that can jeopardize patient safety, trust, and operational continuity. A balanced, proactive approach, combining regulatory compliance, risk mitigation, staff training, and technological safeguards, is essential to protect sensitive data and ensure system resilience. Ultimately, cybersecurity is integral to sustaining patient-centered care in an increasingly digital healthcare ecosystem.

## References

[1] Ronquillo JG, Erik Winterholler J, Cwikla K, Szymanski R, Levy C (2018). Health IT, hacking, and cybersecurity: national trends in data breaches of protected health information. JAMIA Open.

[2] Argaw ST, Troncoso-Pastoriza JR, Lacey D (2020). Cybersecurity of hospitals: discussing the challenges and working towards mitigating the risks. BMC Med Inform Decis Mak.

[3] Biasin E, Kamenjašević E (2022). Cybersecurity of medical devices: new challenges arising from the AI Act and NIS 2 Directive proposals. Int Cybersecur Law Rev.

[4] Wasserman L, Wasserman Y (2022). Hospital cybersecurity risks and gaps: review (for the non-cyber professional). Front Digit Health.

[5] AlZubi AA, Al-Maitah M, Alarifi A (2021). Cyber-attack detection in healthcare using cyber-physical system and machine learning techniques. Soft Comput.

[6] Yusif S, Hafeez-Baig A (2021). A: A conceptual model for cybersecurity governance. J Appl Secur Res.

[7] Abraham C, Chatterjee D, Sims RR (2019). Muddling through cybersecurity: insights from the US healthcare industry. Bus Horiz.

[8] Fausett C, Christovich M, Parker J, Baker J, Keebler J (2021). Telemedicine security: challenges and solutions. Proc Int Symp Hum Factors Ergon Healthc.

[9] Offner KL, Sitnikova E, Joiner K, MacIntyre CR (2021). Towards Understanding Cybersecurity Capability in Australian Healthcare Organisations: A Systematic Review of Recent Trends, Threats and Mitigation. Health security intelligence.

[10] Klindienst J, Ayanian S, Schlegelmilch J, Akselrod H (2022). Preparing for compounding crises: staff shortages and cyber-attack vulnerability in the era of COVID-19. J Bus Contin Emer Plan.

[11] Keshta I, Odeh A (2021). Security and privacy of electronic health records: concerns and challenges. Egypt Inform J.

[12] Vimalachandran P, Liu H, Lin Y, Ji K, Wang H, Zhang Y (2020). Improving accessibility of the Australian My Health Records while preserving privacy and security of the system. Health Inf Sci Syst.

[13] Thuraisingham DM, Khomo FK (2023). Protecting IoT infrastructure: understanding vulnerabilities, threats, and intruders in cybersecurity.

[14] Selvaraj S, Sundaravaradhan S (2020). Challenges and opportunities in IoT healthcare systems: a systematic review. SN Appl Sci.

[15] Rajan Jeyaraj P, Nadar ER (2022). Smart-monitor: patient monitoring system for IoT-based healthcare system using deep learning. IETE J Res.

[16] Sardi A, Rizzi A, Sorano E, Guerrieri A (2020). Cyber risk in health facilities: a systematic literature review. Sustainability.

[17] Lehto M (2022). Cyber-attacks against critical infrastructure. InCyber security: critical infrastructure protection.

[18] Ghelani D (2022). Cyber security, cyber threats, implications and future perspectives: a review.

[19] Tully J, Selzer J, Phillips JP, O'Connor P, Dameff C (2020). Healthcare challenges in the era of cybersecurity. Health Secur.

[20] Nyakasoka L, Naidoo R (2022). Barriers to dynamic cybersecurity capabilities in healthcare software services. EPiC Ser Comput.

[21] Garcia-Perez A, Cegarra-Navarro JG, Sallos MP, Martinez-Caro E, Chinnaswamy A (2023). Resilience in healthcare systems: cyber security and digital transformation. Technovation.

[22] Ahmed NB, Daclin N, Olivaux M, Dusserre G (2023). Cybersecurity challenges for field hospitals: impacts of emergency cyberthreats during emergency situations. Int J Emerg Manag.

[23] Bhuyan SS, Kabir UY, Escareno JM (2020). Transforming healthcare cybersecurity from reactive to proactive: current status and future recommendations. J Med Syst.

[24] Kabir UY, Ezekekwu E, Bhuyan SS, Mahmood A, Dobalian A (2020). Trends and best practices in health care cybersecurity insurance policy. J Healthc Risk Manag.

[25] Prasad G, Gujjar P, Kumar HN, Kumar MA, Chandrappa S (2023). Advances of Cyber Security in the Healthcare Domain for Analyzing Data. Cyber trafficking, threat behavior, and malicious activity monitoring for healthcare organizations.

[26] O’Brien N, Ghafur S, Durkin M (2021). Cybersecurity in health is an urgent patient safety concern: we can learn from existing patient safety improvement strategies to address it. J Patient Saf Risk Manag.

[27] Kilovaty I (2022). Cybersecuring the pipeline. Hous L Rev.

[28] Anand CS, Shanker R (2023). Zero trust resilience strategy for linux crypto ransomware obviation and recuperation. 2023 3rd International Conference on Intelligent Technologies (CONIT).

[29] Barach P, Fisher SD, Adams MJ, Burstein GR, Brophy PD, Kuo DZ, Lipshultz SE (2020). Disruption of healthcare: will the COVID pandemic worsen non-COVID outcomes and disease outbreaks?. Prog Pediatr Cardiol.

[30] Arogundade O (2023). Network security concepts, dangers, and defense best practical. Comp Eng Intell Syst.

[31] Javaid M, Haleem A, Singh RP, Suman R (2023). Towards insighting cybersecurity for healthcare domains: a comprehensive review of recent practices and trends. Cyber Secur Appl.

[32] Mwiinga P Investigating the far-reaching consequences of cybercrime.

[33] Shukla S, George JP, Tiwari K, Kureethara JV (2022). Data ethics and challenges.

[34] Siddiqi MA, Pak W, Siddiqi MA (2022). A study on the psychology of social engineering-based cyberattacks and existing countermeasures. Appl Sci.

[35] Kioskli K, Fotis T, Nifakos S, Mouratidis H (2023). The importance of conceptualising the human-centric approach in maintaining and promoting cybersecurity-hygiene in healthcare 4.0. Appl Sci.

[36] Krittanawong C, Rogers AJ, Johnson KW, Wang Z, Turakhia MP, Halperin JL, Narayan SM (2021). Integration of novel monitoring devices with machine learning technology for scalable cardiovascular management. Nat Rev Cardiol.

[37] Singh AP, Berman AT, Marmarelis ME (2020). Management of lung cancer during the COVID-19 pandemic. JCO Oncol Pract.

[38] Ali RF, Dominic PD, Ali SE, Rehman M, Sohail A (2021). Information security behavior and information security policy compliance: a systematic literature review for identifying the transformation process from noncompliance to compliance. Appl Sci.

[39] Božić V Intelligent and agile risk management. https://www.researchgate.net/profile/Velibor-Bozic-2/publication/372345560_Intelligent_and_Agile_Risk_Management/links/64b1266ac41fb852dd70bb2a/Intelligent-and-Agile-Risk-Management.pdf.

[40] Bozdag A (2023). AIsmosis and the pas de deux of human-AI interaction: exploring the communicative dance between society and artificial intelligence. Online J Commun Media Technol.

[41] Bernot A (2022). Surveillance state: inside China’s quest to launch a new era of social control. By Josh Chin and Liza Lin (St Martin’s Press, 2022, 336pp, $29.99 hbk). The British Journal of Criminology.

[42] Verde D, Paiva S, Lopes S (2023). Assessing cybersecurity risks in BLE-based asset management systems. 2023 30th IWSSIP.

[43] Kumar M, Raj H, Chaurasia N, Gill SS (2023). Blockchain inspired secure and reliable data exchange architecture for cyber-physical healthcare system 4.0. IoT Cyber-Phys Syst.

[44] Wang G, Badal A, Jia X (2022). Development of metaverse for intelligent healthcare. Nat Mach Intell.

[45] Djenna A, Harous S, Saidouni DE (2021). Internet of things meet internet of threats: new concern cyber security issues of critical cyber infrastructure. Appl Sci.

[46] Zhao Y, Wu L (2022). Research on emergency response policy for public health emergencies in China-based on content analysis of policy text and PMC-index model. Int J Environ Res Public Health.

[47] Taheri S, Asadizanjani N (2022). An overview of medical electronic hardware security and emerging solutions. Electronics.

[48] Madavarapu J (2023). Electronic data interchange analysts strategies to improve information security while using EDI in healthcare organizations.

[49] Teichmann F, Boticiu SR, Sergi BS (2023). The evolution of ransomware attacks in light of recent cyber threats. How can geopolitical conflicts influence the cyber climate?. Int Cybersecur Law Rev.

[50] Schiliro F (2023). Building a resilient cybersecurity posture: a framework for leveraging prevent, detect and respond functions and law enforcement collaboration. arXiv [PREPRINT].

[51] Thomasian N, Adashi E (2021). Cybersecurity in the internet of medical things. Health Pol Technol.

[52] Crawford E, Bobrow A, Sun L (2023). Cyberbiosecurity in high-containment laboratories. Front Bioeng Biotechnol.

[53] Akter S, Michael K, Uddin MR, McCarthy G, Rahman M (2022). Transforming business using digital innovations: the application of AI, blockchain, cloud and data analytics. Ann Oper Res.

[54] Weber K, Kleine N (2020). Cybersecurity in Health Care. The ethics of cybersecurity.

[55] Iyer V, Yang Z, Ko J, Weissleder R, Issadore D (2022). Advancing microfluidic diagnostic chips into clinical use: a review of current challenges and opportunities. Lab Chip.

[56] Philip NY, Rodrigues JJ, Wang H, Fong SJ, Chen J (2021). Internet of Things for in-home health monitoring systems: current advances, challenges and future directions. IEEE J Sel Areas Commun.

[57] Jimo S, Abdullah T, Jamal A (2023). IoE Security Risk Analysis in a Modern Hospital Ecosystem. Cybersecurity in the age of smart societies. Advanced sciences and technologies for security applications.

[58] James E, Rabbi F (2023). Fortifying the IoT landscape: strategies to counter security risks in connected systems. Tensorgate J Sust Tech Infra Dev Coun.

[59] Rahman A, Islam MJ, Band SS, Muhammad G, Hasan K, Tiwari P (2023). Towards a blockchain-SDN-based secure architecture for cloud computing in smart industrial IoT. Digit Commun Netw.

[60] Verma A, Bhattacharya P, Madhani N (2022). Blockchain for industry 5.0: vision, opportunities, key enablers, and future directions. IEEE Access.

[61] Roy I, Mitra R, Rahimi N, Gupta B (2023). Efficient non-DHT-based RC-based architecture for fog computing in healthcare 4.0. IoT.

[62] Qu K, Li X, Guo S (2023). Privacy and security in ubiquitous integrated sensing and communication: threats, challenges and future directions. arXiv [PREPRINT].

[63] Soni V, Kukreja D, Sharma DK (2020). Security vs. flexibility: striking a balance in the pandemic era. 2020 IEEE International Conference on Advanced Networks and Telecommunications Systems (ANTS).

[64] Yagi M, Yamanouchi K, Fujita N, Funao H, Ebata S (2023). Revolutionizing spinal care: current applications and future directions of artificial intelligence and machine learning. J Clin Med.

[65] Cascella M, Montomoli J, Bellini V, Vittori A, Biancuzzi H, Dal Mas F, Bignami EG (2023). Crossing the AI chasm in neurocritical care. Computers.

[66] Cacciamani GE, Chen A, Gill IS, Hung AJ (2024). Artificial intelligence and urology: ethical considerations for urologists and patients. Nat Rev Urol.

[67] Ayobi A, Marshall P, Cox AL (2020). Trackly: a customisable and pictorial self-tracking app to support agency in multiple sclerosis self-care. Proceedings of the 2020 CHI Conference on Human Factors in Computing System.

[68] Moustakas E, Lamba N, Mahmoud D, Ranganathan C (2020). Blurring lines between fiction and reality: perspectives of experts on marketing effectiveness of virtual influencers. 2020 International Conference on Cyber Security and Protection of Digital Services (Cyber Security).

[69] Wu M, Luo J (2019). Wearable technology applications in healthcare: a literature review. Online J Nurs Inform.

[70] Abernethy A, Adams L, Barrett M (2022). The promise of digital health: then, now, and the future. NAM Perspect.

[71] Haleem A, Javaid M, Singh RP, Suman R (2022). Medical 4.0 technologies for healthcare: features, capabilities, and applications. IoT Cyber-Phys Syst.

[72] Kugler RL (2009). Deterrence of cyber attacks. Cyberpower and National Security.

[73] Bradford L, Aboy M, Liddell K (2020). COVID-19 contact tracing apps: a stress test for privacy, the GDPR, and data protection regimes. J Law Biosci.

[74] Srinivas J, Das AK, Kumar N (2019). Government regulations in cyber security: framework, standards and recommendations. Future Gener Comput Syst.

[75] Jarjoui S, Murimi R (2021). A Framework for Enterprise Cybersecurity Risk Management. Advances in cybersecurity management.

[76] Nova K (2022). Security and resilience in sustainable smart cities through cyber threat intelligence. Int J Inform Cybersecur.

[77] Walid R, Joshi KP, Choi SG (2021). Secure Cloud EHR with semantic access control, searchable encryption and attribute revocation. 2021 IEEE International Conference on Digital Health (ICDH).

[78] Nagaraju S, Parthiban L (2015). Trusted framework for online banking in public cloud using multi-factor authentication and privacy protection gateway. J Cloud Comput.

[79] Triplett W (2022). Addressing human factors in cybersecurity leadership. J Cybersecur Priv.

[80] Daniel C, Sipper JR (2023). Hacking humans: the art of exploiting psychology in the digital age. J Res Dev.

[81] Ozkaya E (2021). Incident response in the age of cloud: techniques and best practices to effectively respond to cybersecurity incidents. Packt Publishing Ltd, Birmingham, United Kingdom.

[82] Bartock M, Cichonski J, Souppaya M, Witte G, Scarfone K (2016). Guide for cybersecurity event recovery. Natl Inst Stand Technol Spec Publ.

[83] Skopik F, Settanni G, Fiedler R (2016). A problem shared is a problem halved: a survey on the dimensions of collective cyber defense through security information sharing. Comput Secur.

[84] Dhirani LL, Armstrong E, Newe T (2021). Industrial IoT, cyber threats, and standards landscape: evaluation and roadmap. Sensors (Basel).

[85] Medoh C, Telukdarie A (2022). The future of cybersecurity: a system dynamics approach. Procedia Comput Sci.

[86] Nguyen TN (2023). Developing health information systems in developing countries: lessons learnt from a longitudinal action research study in Vietnam. Electron J Inf Syst Dev Ctries.

[87] Swasey K (2020). Insufficient healthcare cybersecurity invites ransomware attacks and sale of phi on the dark web. https://www.usu.edu/cai/files/studentpaper-swasey.pdf.

[88] Minnaar A, Herbig FJ (2021). Cyberattacks and the cybercrime threat of ransomware to hospitals and healthcare services during the COVID-19 pandemic. Acta Crim.

[89] Al-Qarni EA (2023). Cybersecurity in healthcare: a review of recent attacks and mitigation strategies. Int J Adv Comput Sci Appl.

[90] Serna S (2022). The increase of ransomware attacks within the healthcare and education sector. Utica University ProQuest Dissertations Publishing.

[91] Alawida M, Omolara AE, Abiodun OI, Al-Rajab M (2022). A deeper look into cybersecurity issues in the wake of Covid-19: a survey. J King Saud Univ Comput Inf Sci.

[92] Kolevski D, Michael K, Abbas R, Freeman M (2021). Cloud data breach disclosures: the consumer and their personally identifiable information (PII)?. 2021 IEEE Conference on Norbert Wiener in the 21st Century (21CW).

[93] Antwi M, Adnane A, Ahmad F, Hussain R, ur Rehman MH, Kerrache CA (2021). The case of HyperLedger Fabric as a blockchain solution for healthcare applications. Blockchain: Res Appl.

[94] Ghafur S, Kristensen S, Honeyford K, Martin G, Darzi A, Aylin P (2019). A retrospective impact analysis of the WannaCry cyberattack on the NHS. NPJ Digit Med.

[95] Miller-Rushing AJ, Athearn N, Blackford T (2021). COVID-19 pandemic impacts on conservation research, management, and public engagement in US national parks. Biol Conserv.

[96] Bandari V (2023). Enterprise data security measures: a comparative review of effectiveness and risks across different industries and organization types. Int J Bus Intel Big Data Anal.

[97] Kumar S, Bharti AK, Amin R (2021). Decentralized secure storage of medical records using Blockchain and IPFS: a comparative analysis with future directions. Secur Priv.

[98] Jampen D, Gür G, Sutter T, Tellenbach B (2020). Don’t click: towards an effective anti-phishing training. A comparative literature review. Hum Cent Comput Inf Sci.

[99] Telo J (2023). Smart city security threats and countermeasures in the context of emerging technologies. Int J Intell Autom Comput.

[100] Kechagias EP, Chatzistelios G, Papadopoulos GA, Apostolou P (2022). Digital transformation of the maritime industry: a cybersecurity systemic approach. Int J Crit Infrastruct Prot.

[101] Tapia J, Castanho R (2023). Cybersecurity and geopolitics in the Dominican republic: threats, policies and future prospects. Dutch J Financ Manag.

